# Dapagliflozin versus empagliflozin in patients with chronic kidney disease

**DOI:** 10.3389/fphar.2023.1227199

**Published:** 2023-08-04

**Authors:** Hilmi Alnsasra, Gal Tsaban, Adam Solomon, Fouad Khalil, Enis Aboalhasan, Abed N. Azab, Joseph Azuri, Ariel Hammerman, Ronen Arbel

**Affiliations:** ^1^ Department of Cardiology, Soroka University Medical Center, Beersheba, Israel; ^2^ Faculty of Health Sciences, Ben Gurion University of the Negev, Beer-Sheva, Israel; ^3^ Sanford School of Medicine, University of South Dakota, Vermillion, SD, United States; ^4^ Maximizing Health Outcomes Research Lab, Sapir College, Sderot, Israel; ^5^ Department of Nursing, Department of Clinical Biochemistry and Pharmacology, Faculty of Health Sciences, Ben Gurion University of the Negev, Beer-Sheva, Israel; ^6^ Diabetes Clinic, Maccabi Healthcare Services, Tel Aviv, Israel; ^7^ Sackler Faculty of Medicine, Tel Aviv University, Tel Aviv, Israel; ^8^ Department of Pharmaceutical Technology Assessment, Clalit Health Services Headquarters, Tel Aviv, Israel

**Keywords:** chronic kidney disease, dapagliflozin, empagliflozin, cost needed to treat, outcomes

## Abstract

**Background and Aim:** Dapagliflozin and empagliflozin have demonstrated favorable clinical outcomes among patients with chronic kidney disease (CKD). However, their comparative monetary value for improving outcomes in CKD patients is unestablished. We examined the cost-per-outcome implications of utilizing dapagliflozin as compared to empagliflozin for prevention of renal and cardiovascular events in CKD patients.

**Methods:** For calculation of preventable events we divided the allocated budget by the cost needed to treat (CNT) for preventing a single renal or cardiovascular event. CNT was derived by multiplying the annualized number needed to treat (aNNT) by the annual therapy cost. The aNNTs were determined based on data from the DAPA-CKD and EMPEROR-KIDNEY trials. The budget limit was defined based on the threshold recommended by the United States’ Institute for Clinical and Economic Review.

**Results:** The aNNT was 42 both dapagliflozin (95% confidence interval [CI]: 34-59) and empagliflozin (CI: 33-66). The CNT estimates for the prevention of one primary event for dapagliflozin and empagliflozin were comparable at $201,911 (CI: $163,452-$283,636) and $209,664 (CI: $164,736-$329,472), respectively. However, diabetic patients had a higher CNT with dapagliflozin ($201,911 [CI: $153,837-$346,133]) than empagliflozin ($134,784 [CI: $109,824-$214,656]), whereas non-diabetic patients had lower CNT for dapagliflozin ($197,103 [CI: $149,029-$346,133]) than empagliflozin ($394,368 [CI: $219,648-$7,093,632]). The CNT for preventing CKD progression was higher for dapagliflozin ($427,858 [CI: $307,673-$855,717]) than empagliflozin ($224,640 [CI: $169,728-$344,448]). For preventing cardiovascular death (CVD), the CNT was lower for dapagliflozin ($1,634,515 [CI: $740,339-∞]) than empagliflozin ($2,990,208 [CI: $1,193,088-∞]).

**Conclusion:** Among patients with CKD, empagliflozin provides a better monetary value for preventing the composite renal and cardiovascular events in diabetic patients while dapagliflozin has a better value for non-diabetic patients. Dapagliflozin provides a better monetary value for the prevention of CVD, whereas empagliflozin has a better value for the prevention of CKD progression.

## 1 Introduction

Chronic kidney disease (CKD) typically exhibits a progressive course leading to diminished quality of life and reduced life expectancy (Webster et al., 2017). The progressive nature of CKD and the need for kidney-replacement therapy pose an extreme financial burden on health systems worldwide (Webster et al., 2017).

Notably, sodium–glucose cotransporter 2 (SGLT2) inhibitors have shown favorable effects on kidney and cardiovascular outcomes in large-scale clinical trials involving patients with type 2 diabetes (Wanner et al., 2016; Neal et al., 2017; Perkovic et al., 2019; Wiviott et al., 2019). The DAPA-CKD trial included participants with an estimated glomerular filtration rate (eGFR) of 25–75 mL/min/1.73 m^2^, concluding that regardless of diabetic status, the risk of a composite of a sustained decline in the eGFR of at least 50%, end-stage kidney disease, or death from renal or cardiovascular causes was significantly lower with dapagliflozin than with placebo (Heerspink et al., 2020). More recently, the EMPA-KIDNEY trial showed that among a wide range of patients with CKD (eGFR of 20–90 mL/min/1.73 m^2^), empagliflozin therapy led to a lower risk of progression of CKD or death from cardiovascular causes than placebo regardless of diabetic status (The EMPA-KIDNEY Collaborative Group Herrington et al., 2023).

Based on the rapidly emerging new evidence of SGLT2 inhibitors’ efficacy and safety in CKD, the Kidney Disease Improving Global Outcomes (KDIGO) guidelines and The National Institute of Health and Care Excellence (NICE) clinical guidelines on CKD recommend SGLT2 inhibitors for patients with CKD and type 2 diabetes (NICE Guideline, 2021; Rossing et al., 2022).

Despite the results of these promising large-scale clinical trials, a recently published analysis suggests that SGLT2 inhibitors remain underutilized in patients with CKD, especially in patients without diabetes mellitus (DM) (Zhuo et al., 2022). An additional recent analysis showed low utilization rates of SGLT2 inhibitors in patients with atherosclerotic cardiovascular disease, heart failure and type 2 DM (Hussain et al., 2023). The prices of these medications play a major role in this challenge and may preclude routine prescription and decrease patients’ adherence (Aggarwal et al., 2022). In this context, several studies have shown that decreasing patients’ out-of-pocket costs of medication increases their adherence to pharmacotherapy and upsurges physicians’ prescription rate (Robinson et al., 2020). To the best of our knowledge, a comparative cost-per-outcome analysis between dapagliflozin and empagliflozin in CKD is lacking. To assist decision-makers in prioritizing SGLT2 inhibitors, we aimed to compare the cost-per-outcome implications of dapagliflozin and empagliflozin in patients with CKD in the presence or absence of DM based on the results of the DAPA-CKD and EMPA-KIDNEY clinical trials under a predefined budget constraint that was set by the Institute for Clinical and Economic Review (ICER) (Lin et al., 2022).

## 2 Material and methods

### 2.1 Data sources for drug efficacy

Outcome data for dapagliflozin and empagliflozin were extracted from the DAPA-CKD and EMPA-KIDNEY trials, respectively (Heerspink et al., 2020; The EMPA-KIDNEY Collaborative Group Herrington et al., 2023).

### 2.2 Outcome measures

Our main study outcome was the cost needed to treat (CNT) to prevent one event of the primary outcome, as defined in each trial in the total population and stratified by diabetic status (Heerspink et al., 2020; The EMPA-KIDNEY Collaborative Group Herrington et al., 2023). The secondary outcomes used for the analysis were similarly derived from the DAPA-CKD and EMPA-KIDNEY trials. Secondary outcomes were the CNT to prevent one event of CKD progression, cardiovascular death (CVD), or all-cause mortality as separate clinical outcomes.

### 2.3 CNT analysis

The number of preventable primary renal or cardiovascular events achievable with dapagliflozin or empagliflozin was estimated by dividing the predefined maximum available budget by the CNT to prevent one event. The budget limit, $734 million, was set as the United States’ threshold suggested by the ICER (Lin et al., 2022). The CNT was determined as the product of the annualized number needed to treat (aNNT) to prevent a single event multiplied by the cost of therapy (Mayne et al., 2006). Analysis was performed from the United States healthcare payer perspectives. Drug costs were calculated as 75% of the US National Average Drug Acquisition Cost (NADAC), extracted in July 2022 (Pharmacy Pricing, 2022).

### 2.4 aNNT analysis

The aNNT was derived by dividing one by the annualized absolute risk reduction (aARR). The aARR was determined as the absolute difference between the annualized absolute risk (aAR) observed in the control arm and the treatment arm. To calculate the aAR, the number of events in each study arm was divided by the patient-years of treatment (Mayne et al., 2006). The event rates for this analysis were based on the CKD adjudication criteria defined in the original trials.

### 2.5 Sensitivity analysis

We conducted a one-way sensitivity analyses in order to mitigatedifferences in baseline risk among the different randomized controlled trials (RCT) populations. Accounting for parameters that may affect the NNT and CNT figures (Mendes et al., 2017). Two parameters were included: the risk of events in the RCTs’ control arm and the compared annual costs of the interventions. For the sensitivity analysis we employed the full NADAC price as the upper limit and 50% of the NADAC price as the lower limit, This approach aligns with the recommended methodology for conducting US cost-effectiveness analyses (Levy et al., 2018).

## 3 Results

### 3.1 Patient populations

A total of 10,913 patients were included in the two randomized trials (Heerspink et al., 2020; The EMPA-KIDNEY Collaborative Group Herrington et al., 2023), as presented in Table 1. The median follow-up time was longer in DAPA-CKD (2.4 years) compared to EMPA-KIDNEY (2 years). Overall, the DAPA-CKD trial included patients with an eGFR of 25–75 mL/min/1.73 m^2^ and urinary albumin-to-creatinine ratio of 200–5,000 compared to an eGFR of 20–45 mL/min/1.73 m^2^ and urinary albumin-to-creatinine ratio of at least 200 for the EMPA-KIDNEY trial (Table 1). Both trials required patients to take a renin-angiotensin system (RAS) inhibitor; however, only the DAPA-CKD trial specified the required drug classes. Namely, the documented inability to take an angiotensin-converting enzyme inhibitor (ACEi) or angiotensin receptor blocker (ARB) was an exclusion criterion for the DAPA-CKD trial, while this was not the case for the EMPA-KIDNEY trial (Heerspink et al., 2020; The EMPA-KIDNEY Collaborative Group Herrington et al., 2023).

**TABLE 1:** Baseline characteristics.

	DAPA-CKD		EMPA-KIDNEY	
Characteristic	Dapagliflozin (N = 2,152)	Placebo (N = 2,152)	Empagliflozin (N = 3,304)	Placebo (N = 3,305)
Age, yr	61.8 ± 12.1	61.9 ± 12.1	63.9 ± 13.9	63.8 ± 13.9
Female sex- no. (%)	709 (32.9)	716 (33.3)	1,097 (33.2)	1,095 (33.1)
Race, no. (%)				
White	1,124 (52.2)	1,166 (54.2)	1939 (58.7)	1920 (58.1)
Black	104 (4.8)	87 (4.0)	128 (3.9)	134 (4.1)
Type 2 diabetes, no. (%)	1,455 (67.6)	1,451 (67.4)	1,470 (44.5)	1,466 (44.4)
Cardiovascular disease, no. (%)	813 (37.8)	797 (37.0)	861 (26.1)	904 (27.4)
BMI	29.4 ± 6.0	29.6 ± 6.3	29.7 ± 6.7	29.8 ± 6.8
Estimated GFR (mean)	43.2 ± 12.3	43.0 ± 12.4	37.4 ± 14.5	37.3 ± 14.4
Urinary albumin-to-creatinine ratio, median (IQR)	965 (472–1903)	934 (482–1868)	331 (46–1,061)	327 (54–1,074)

### 3.2 Study outcomes

**FIGURE 1:**
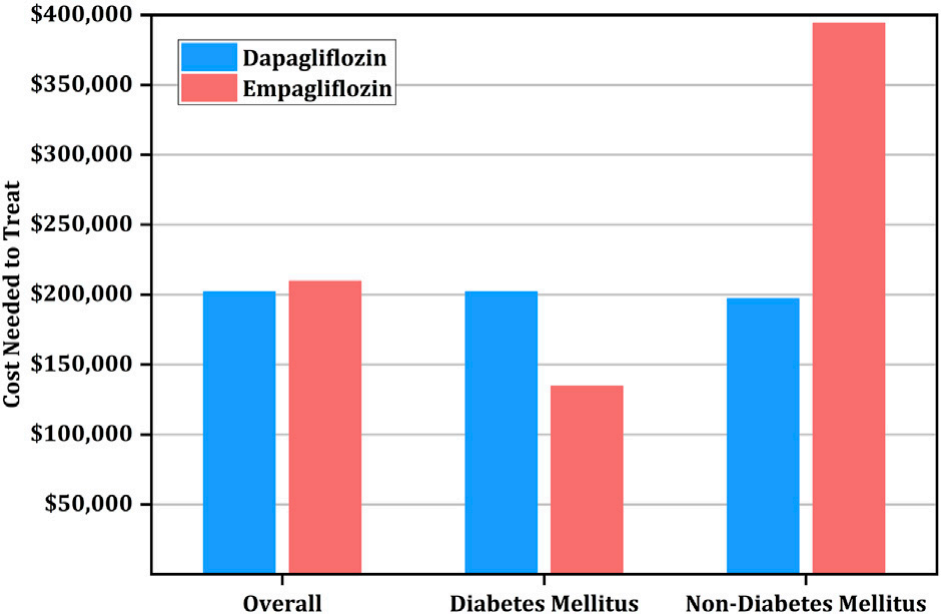
Cost needed to treat (CNT) for prevention of primary outcome using empagliflozin versus dapagliflozin stratified by diabetic status.

The aNNT with both dapagliflozin and empagliflozin was 42, with a 95% confidence interval (CI) of 34–59 and 33-66, respectively (Table 2). Since the annual drug costs are $4,807 and $4,992 for dapagliflozin and empagliflozin, respectively; the overall CNT to prevent one event of CKD is $201,911 (95% CI: $163,452-$283,636) for dapagliflozin versus $209,664 (95% CI: $164,736-$329,472) for empagliflozin (Table 2; Figure 1).

**TABLE 2:** Step-by-step calculations of the number and cost needed to treat for empagliflozin and dapagliflozin.

	Dapagliflozin		Empagliflozin	
Trial	DAPA-CKD		EMPA-KIDNEY	
Group	Control	Intervention	Control	Intervention
Follow-up (years)	2.4		2	
Patient number	2,152	2,152	3,305	3,304
Patient years	5,165	5,165	6,610	6,608
Events	312	190	558	402
Annualized event rate	6.04%	3.68% (3.08%–4.35%)	8.44%	6.08% (5.40%–6.92%)
Annualized Absolute Event Rate Reduction (95% CI)	2.36% (1.69%–2.96%)		2.36% (1.52%–3.04%)	
Annualized Number Needed to Treat (95% CI)	42 (34–59)		42 (33–66)	
Annual drug cost	$4,807		$4,992	
Cost Needed to Treat to prevent one event (95% CI)	$201,911 ($163,452-$283,636)		$209,664 ($164,736-$329,472)	

CI, confidence interval.

If the annual predefined budget of $734 million was allocated entirely for the prevention of renal and cardiovascular events, a similar number of events would be prevented using dapagliflozin (2,726 events, [95% CI: 941-3,368]) and empagliflozin (2,626 events, [95% CI:1,671-3,342]) when utilizing the US high-cost estimate (Table 3).

**TABLE 3:** Avoided primary renal and cardiovascular events in low and high cost estimates of empagliflozin and dapagliflozin.

Price estimate	US (low estimate)		US (high estimate)	
Treatment	Dapagliflozin	Empagliflozin	Dapagliflozin	Empagliflozin
Annual Cost	$3,205	$3,328	$6,410	$6,656
Annual Budget	$ 734,000,000			
CNT (95% CI)	$134,610 ($108,970-$189,095)	$139,776 ($109,824-$219,648)	$269,220 ($217,940-$378,190)	$279,552 ($219,648-$439,296)
Prevented events within the budget	5,453 (3,882-6,736)	5,251 (3,342-6,683)	2,726 (1,941-3,368)	2,626 (1,671-3,342)
N (95% CI)				

CI, confidence interval; CNT, cost needed to treat.

Of note, among diabetic patients, the CNT was higher for dapagliflozin at $201,911 (95% CI: $153,837-$346,133) than empagliflozin at $134,784 (95% CI: $109,824-$214,656). However, the CNT of dapagliflozin was lower than empagliflozin among patients without diabetes at $197,103 (95% CI: $149,029-$346,133) versus $394,368 (95% CI: $219,648-$7,093,632) (Table 4; Figure 1).

**TABLE 4:** Cost needed to treat for the prevention of the primary outcome in diabetic and non-diabetic patients.

	Dapagliflozin	Empagliflozin
Diabetic (95% CI)	$201,911 ($153,837- $346,133)	$134,784 ($109,824- $214,656)
Non-diabetic (95% CI)	$197,103 ($149,029- $346,133)	$394,368 ($219,648- $7,093,632)

CI, confidence interval.

**FIGURE 2:**
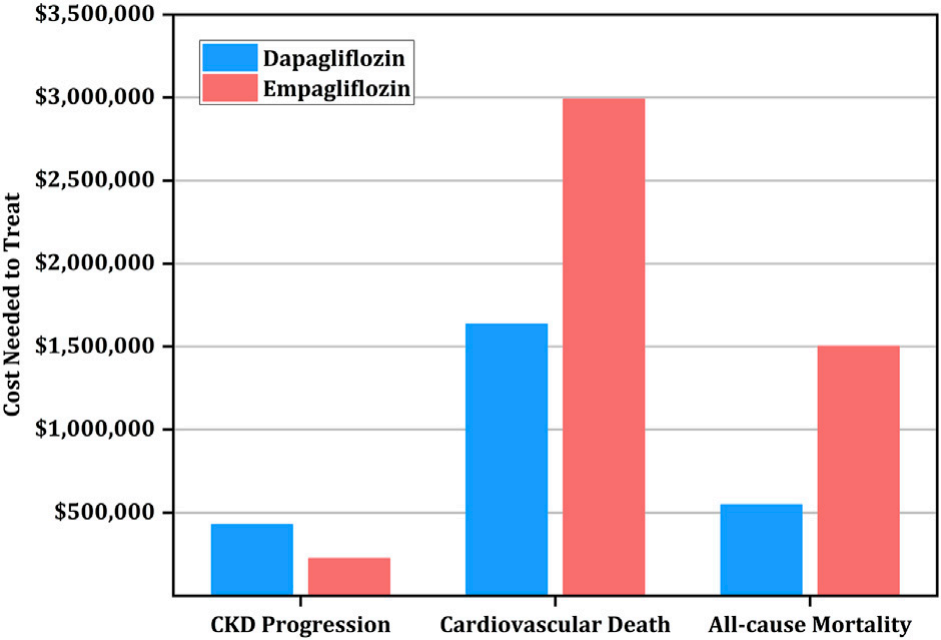
Cost needed to treat (CNT) for prevention of secondary outcomes using empagliflozin versus dapagliflozin.

Interestingly, the drugs have varying effects on reducing secondary outcomes. Specifically, the CNT for reducing the progression of CKD was higher for dapagliflozin ($427,858 [95% CI: $307,673-$855,717]) than empagliflozin ($224,640 [95% CI: $169,728-$344,448]), while the CNT for CVD and all-cause mortality prevention was higher for empagliflozin ($2,990,208, [$1,193,088-∞]) versus ($1,634,515, [$740,339-∞]) and ($1,502,592 [$648,960-∞]) versus ($548,043 [$360,555-$1,418,182]), respectively (Table 5; Figure 2).

**TABLE 5:** Cost needed to treat for prevention of secondary outcomes.

	Dapagliflozin	Empagliflozin
Progression of CKD (95% CI)	$427,858 ($307,673-$855,717)	$224,640 ($169,728-$344,448)
Cardiovascular Death (95% CI)	$1,634,515 ($740,339-∞)	$2,990,208 ($1,193,088-∞)
All-cause Mortality (95% CI)	$548,043 ($360,555-$1,418,182)	$1,502,592 ($648,960-∞)

CI, confidence interval; CKD, chronic kidney disease.

## 4 Discussion

Our analysis suggests that dapagliflozin and empagliflozin provide similar NNTs for preventing one primary event in CKD. Overall, the monetary values of dapagliflozin and empagliflozin were similar. However, the CNT was lower for empagliflozin in diabetic patients but higher than dapagliflozin in patients without DM. Additionally, we found that CNT analysis has varying results on secondary outcomes, with empagliflozin ensuing a better monetary value for the reduction of CKD progression and dapagliflozin displayed a better monetary value for preventing CVD and all-cause mortality.

EMPA-KIDNEY included 6,609 participants who were randomized to either empagliflozin or placebo (The EMPA-KIDNEY Collaborative Group Herrington et al., 2023). The primary outcome of progression of kidney disease or CVD was 13.1% with empagliflozin versus 16.9% with placebo. These outcomes were consistent regardless of diabetic status or prior cardiovascular disease. DAPA-CKD included 4,304 patients with CKD where the primary outcome (a composite of a sustained decline in the eGFR of at least 50%, end-stage kidney disease, or death from renal/cardiovascular causes) occurred in 9.2% in the dapagliflozin group and 14.5% in the placebo group (Heerspink et al., 2020).

McEwan et al. evaluated the potential long-term economic effect of dapagliflozin in CKD treatment (McEwan et al., 2022). Their study illustrated the cost-effectiveness of dapagliflozin versus placebo using patient-level data from the DAPA-CKD trial. Results were consistent across Germany, Spain and the United Kingdom with incremental cost-effectiveness ratios of $17,623, $11,687, and $8,280, respectively (McEwan et al., 2022). This finding was also in line with a recent study in the US that demonstrated the cost-effectiveness of dapagliflozin in non-diabetic CKD patients relative to a willingness-to-pay threshold of $100,000 per quality-adjusted life years (QALY) gained. The study showed that adding dapagliflozin increased life expectancy by 2 years compared to standard therapy alone, along with an additional 1.3 discounted QALY. Furthermore, dapagliflozin provided a $79,000 increase in the total discounted lifetime healthcare costs. The study indicated that the net 1-year budgetary cost of treating all non-diabetic CKD patients in the US could be up to $21 billion (Tisdale et al., 2022). Similarly, another study illustrated the cost-effectiveness of dapagliflozin for patients with stage 3b CKD in Japan (Kodera et al., 2022). A very recent analysis showed that the use of canagliflozin or dapagliflozin, added to the standard of care (SoC) in patients with CKD and DM, was more cost-effective than SoC only (Nguyen et al., 2023).

Although empagliflozin has demonstrated cost-effectiveness in different patient populations, less is known about its cost-effectiveness for CKD patients compared to dapagliflozin. However, a cost-effective analysis based on results from the EMPA-REG OUTCOME study demonstrated an incremental cost-effective ratio of $25,974 per QALY with the addition of empagliflozin compared to standard therapy alone in the US (Reifsnider et al., 2022). Noteworthy, the EMPA-REG OUTCOME study was not powered to assess benefits in the diabetic CKD subgroup, in addition to excluding patients with an eGFR of <30 mL/min/1.73 m^2^ (Zinman et al., 2015). A recent pooled analysis of EMPEROR-Reduced and EMPEROR-Preserved (Empagliflozin Outcome Trial in Patients with Chronic Heart Failure with Reduced or Preserved Ejection Fraction, respectively) demonstrated that the benefit of empagliflozin on heart failure events was not influenced by KDIGO categories based on eGFR and urine albumin-to-creatinine ratio (Butler et al., 2023). Moreover, recent data demonstrated that the effects of the new renal protective medications, including SGLT2 inhibitors, remain consistent using different eGFR decline thresholds (Heerspink et al., 2023). However, the EMPA-KIDNEY trial showed no convincing evidence justifying the use of empagliflozin in patients with chronic kidney disease without macroalbuminuria (Herrington et al., 2023).

SGLT2-inhibitors have shown several clinical benefits beyond diabetes, including heart failure (HF), and CKD. It was recently indicated that empagliflozin improves frailty in elderly patients with DM and hypertension by mitigating oxidative stress in endothelial cells (Santulli et al., 2023). An additional recent report showed that empagliflozin mediates the modification of microRNAs that are involved in the regulation of endothelial function in diabetic patients with heart failure with preserved left ventricular function (Mone et al., 2023).

In addition to the differences in the reported clinical outcomes of empagliflozin and dapagliflozin, it is worth noting that these medications also confer various pharmacological properties that may influence their therapeutic benefits. For example, empagliflozin is more selective for SGLT2 over SGLT1 than dapagliflozin (Grempler et al., 2012). Expression of SGLT1 in the heart is markedly elevated, in contrast to its comparably diminished expression in the kidney (Zhou et al., 2003). Conversely, SGLT2 exhibits very high expression in the kidney, while significantly reduced in the heart (Zhou et al., 2003). Thus, the much higher selectivity of empagliflozin for SGLT2 over SGLT1, in comparison to that of dapagliflozin, may result in a different pharmacological as well as clinical profile. For instance, empagliflozin is expected to alter glucose transport mainly in the kidney while having a negligible effect on glucose uptake in the heart (Anker and Butler, 2018). This could underlie the better value of dapagliflozin for cardiovascular outcomes in non-diabetic patients, as well as of empagliflozin for CKD and in diabetics patients. Accumulating data suggests that SGLT2 inhibitors (particularly dapagliflozin) inhibit neurohormonal activation among patients with systolic dysfunction (Lymperopoulos et al., 2021; Koufakis et al., 2022; Udell et al., 2022; Talha et al., 2023). It has been suggested that these medications mitigate the activity of the sympathetic nervous system, leading to a reduction in catecholamines secretion from the adrenal medulla and suppression of the activation of the renin-angiotensin-aldosterone system. Altogether, these effects may contribute to improvement of endothelial function, prevention of left ventricular remodeling, fibrosis and HF. (Udell et al., 2022).

Although a growing body of evidence supports the role of SGLT2 inhibitors as disease-modifying agents for CKD, their use is still limited in clinical practice partially due to their cost (Aggarwal et al., 2022). Our analysis attempts to provide some cost-per-outcome insight when prescribing SGLT2 inhibitors to CKD patients. To our knowledge, this is the first cost-per-outcome comparison between empagliflozin and dapagliflozin in patients with CKD. Our results suggest variability of CNT according to patients’ characteristics and certain secondary outcomes. Diabetic status seems to be an important factor to consider when choosing between these medications to achieve the best monetary value per outcome. Future studies are needed to confirm these findings.

### 4.1 Limitations

Several limitations should be acknowledged in our study. Firstly, it is important to recognize that our analysis does not supplant the necessity for a thorough cost-effectiveness assessment in relation to QALY. Furthermore, this analysis did not encompass the entirety of relevant data required for a formal cost-effective analysis, including factors such as expenses and benefits associated with healthcare services beyond the therapy itself. These considerations include adverse effects, hospitalization rates, and quality of life measures. However, given the absence of such comparative studies with empagliflozin and dapagliflozin in CKD patients, our CNT analysis could serve as a prompt tool for comparative monetary value. Moreover, CNT provides an actual, real-world comparison of the costs needed to prevent one primary event in patients with CKD. That way, CNT gives a practical insight into monetary value for those in the healthcare field in a position to make healthcare decisions for patients. Inherent limitations of the trials used in our analysis must also be considered, especially the differences in populations of the two trials. Our sensitivity analysis attempts to overcome these differences by simulating each drug’s effect in each RCT.

## 5 Conclusion

In analysing data from the DAPA-CKD and EMPEROR-KIDNEY trials, the CNT to prevent one primary event was lower for empagliflozin than dapagliflozin in patients with DM, but lower for dapagliflozin than empagliflozin among patients without DM. Moreover, the CNT to prevent CKD progression was lower for empagliflozin, but lower for dapagliflozin to prevent CVD and all-cause mortality.

## Data Availability

The raw data supporting the conclusion of this article will be made available by the authors, without undue reservation.

## References

[B1] Aggarwal R. Vaduganathan M. Chiu N. Bhatt D. L. (2022). Out-of-Pocket costs for SGLT-2 (Sodium-Glucose transport protein-2) inhibitors in the United States. Circ. Heart Fail 15 (3), e009099. 10.1161/CIRCHEARTFAILURE.121.009099 34886682 PMC8920018

[B2] Anker S. D. Butler J. (2018). Empagliflozin, calcium, and SGLT1/2 receptor affinity: Another piece of the puzzle. Esc. Heart Fail 5, 549–551. 10.1002/ehf2.12345 30024112 PMC6073022

[B3] Butler J. Packer M. Siddiqi T. J. Böhm M. Brueckmann M. Januzzi J. L. (2023). Efficacy of empagliflozin in patients with heart failure across kidney risk categories. J. Am. Coll. Cardiol. 81 (19), 1902–1914. 10.1016/j.jacc.2023.03.390 37164523

[B4] Grempler R. Thomas L. Eckhardt M. Himmelsbach F. Sauer A. Sharp D. E. (2012). Empagliflozin, a novel selective sodium glucose cotransporter-2 (SGLT-2) inhibitor: Characterisation and comparison with other SGLT-2 inhibitors. Diabetes Obes. Metab. 14, 83–90. 10.1111/j.1463-1326.2011.01517.x 21985634

[B5] Heerspink H. J. L. Stefánsson B. V. Correa-Rotter R. Chertow G. M. Greene T. Hou F. F. (2020). Dapagliflozin in patients with chronic kidney disease. N. Engl. J. Med. 383 (15), 1436–1446. 10.1056/nejmoa2024816 32970396

[B6] Heerspink H. J. L. Jongs N. Neuen B. L. Schloemer P. Vaduganathan M. Inker L. A. (2023). Effects of newer kidney protective agents on kidney endpoints provide implications for future clinical trials. Kidney Int. 104, 181–188. 10.1016/j.kint.2023.03.037 37119876

[B8] Herrington W. G. Baigent C. Haynes R. (2023). Empagliflozin in patients with chronic kidney disease. Reply. N. Engl. J. Med. 388, 2301–2302. 10.1056/NEJMc2301923 37314723

[B9] Hussain A. Ramsey D. Lee M. Mahtta D. Khan M. S. Nambi V. (2023). Utilization rates of SGLT2 inhibitors among patients with type 2 diabetes, heart failure, and atherosclerotic cardiovascular disease: Insights from the department of veterans affairs. JACC Heart Fail. S2213-1779(23)00185-3. [Epub ahead of print]. 10.1016/j.jchf.2023.03.024 37204363

[B10] Kodera S. Morita H. Nishi H. Takeda N. Ando J. Komuro I. (2022). Cost-effectiveness of dapagliflozin for chronic kidney disease in Japan. Circ. J. 86 (12), 2021–2028. 10.1253/circj.CJ-22-0086 36070962

[B11] Koufakis T. Giannakoulas G. Zebekakis P. Kotsa K. (2022). The effect of dapagliflozin on ventricular arrhythmias, cardiac arrest, or sudden death in people with heart failure: A tick in another box for sodium-glucose cotransporter 2 inhibitors. Expert Opin. Pharmacother. 23, 321–325. 10.1080/14656566.2021.2003329 34761713

[B12] Levy J. Rosenberg M. Vanness D. (2018). A transparent and consistent approach to assess US outpatient drug costs for use in cost-effectiveness analyses. Value Health 21 (6), 677–684. 10.1016/j.jval.2017.06.013 29909872 PMC6394851

[B13] Lin G. A. Brouwer E. Nikitin D. Moradi A. Chen Y. Herron-Smith S. (2022). Tirzepatide for type 2 diabetes final report. Boston: Institute for Clinical and Economic Review. Available at: https://icer.org/assessment/diabetes-type-2-2022/#timeline .

[B14] Lymperopoulos A. Borges J. I. Cora N. Sizova A. (2021). Sympatholytic mechanisms for the beneficial cardiovascular effects of SGLT2 inhibitors: A research hypothesis for dapagliflozin’s effects in the adrenal gland. Int. J. Mol. Sci. 22, 7684. 10.3390/ijms22147684 34299304 PMC8305388

[B15] Mayne T. J. Whalen E. Vu A. (2006). Annualized was found better than absolute risk reduction in the calculation of number needed to treat in chronic conditions. J. Clin. Epidemiol. 59 (3), 217–223. 10.1016/j.jclinepi.2005.07.006 16488351

[B16] McEwan P. Darlington O. Miller R. McMurray J. J. V. Wheeler D. C. Heerspink H. J. L. (2022). Cost-effectiveness of dapagliflozin as a treatment for chronic kidney disease: A health-economic analysis of DAPA-CKD. Clin. J. Am. Soc. Nephrol. 17 (12), 1730–1741. 10.2215/CJN.03790322 36323444 PMC9718008

[B17] Mendes D. Alves C. Batel-Marques F. (2017). Number needed to treat (NNT) in clinical literature: An appraisal. BMC Med. 15 (1), 112. 10.1186/s12916-017-0875-8 28571585 PMC5455127

[B18] Mone P. Lombardi A. Kansakar U. Varzideh F. Jankauskas S. S. Pansini A. (2023). Empagliflozin improves the MicroRNA signature of endothelial dysfunction in patients with heart failure with preserved ejection fraction and diabetes. J. Pharmacol. Exp. Ther. 384, 116–122. 10.1124/jpet.121.001251 36549862 PMC9827502

[B19] Neal B. Perkovic V. Mahaffey K. W. de Zeeuw D. Fulcher G. Erondu N. (2017). Canagliflozin and cardiovascular and renal events in type 2 diabetes. N. Engl. J. Med. 377 (7), 644–657. 10.1056/NEJMoa1611925 28605608

[B20] Nguyen B. Mital S. Bugden S. Nguyen H. V. (2023). Cost‐effectiveness of canagliflozin and dapagliflozin for treatment of patients with chronic kidney disease and type 2 diabetes. Diabetes Obes. Metab., 10.1111/dom.15201 37409571

[B21] NICE Guideline (2021). Chronic kidney disease: Assessment and management. London: National Institute for Health and Care Excellence. Available at: https://www.nice.org.uk/guidnace/ng203 . 34672500

[B22] Perkovic V. Jardine M. J. Neal B. Bompoint S. Heerspink H. J. L. Charytan D. M. (2019). Canagliflozin and renal outcomes in type 2 diabetes and nephropathy. N. Engl. J. Med. 380 (24), 2295–2306. 10.1056/NEJMoa1811744 30990260

[B23] Pharmacy Pricing (2022). National average drug acquisition cost. Available at: https://www.medicaid.gov/medicaid/prescription-drugs/pharmacy-pricing/index.html .

[B24] Reifsnider O. S. Kansal A. R. Wanner C. Pfarr E. Koitka-Weber A. Brand S. B. (2022). Cost-effectiveness of empagliflozin in patients with diabetic kidney disease in the United States: Findings based on the EMPA-REG OUTCOME trial. Am. J. Kidney Dis. 79 (6), 796–806. 10.1053/j.ajkd.2021.09.014 34752913

[B25] Robinson J. C. Whaley C. Brown T. T. Dhruva S. S. (2020). Physician and patient adjustment to reference pricing for drugs. JAMA Netw. Open 3 (2), e1920544. 10.1001/jamanetworkopen.2019.20544 32022881 PMC12549095

[B26] Rossing P. Caramori M. L. Chan J. C. N. Heerspink H. J. L. Hurst C. Khunti K. (2022). Executive summary of the KDIGO 2022 clinical practice guideline for diabetes management in chronic kidney disease: An update based on rapidly emerging new evidence. Kidney Int. 102 (5), 990–999. 10.1016/j.kint.2022.06.013 36272755

[B27] Santulli G. Varzideh F. Forzano I. Wilson S. Salemme L. de Donato A. (2023). Functional and clinical importance of SGLT2-inhibitors in frailty: From the kidney to the heart. Hypertension. 10.1161/HYPERTENSIONAHA.123.20598 PMC10529735 37403685

[B28] Talha K. M. Anker S. D. Butler J. (2023). SGLT-2 inhibitors in heart failure: A Review of current evidence. Int. J. heart Fail. 5, 82–90. 10.36628/ijhf.2022.0030 37180562 PMC10172076

[B29] Tisdale R. L. Cusick M. M. Aluri K. Z. Handley T. J. Joyner A. K. C. Salomon J. A. (2022). Cost-effectiveness of dapagliflozin for non-diabetic chronic kidney disease. J. Gen. Intern Med. 37 (13), 3380–3387. 10.1007/s11606-021-07311-5 35137296 PMC9551016

[B7] The EMPA-KIDNEY Collaborative Group Herrington W. G. Staplin N. Wanner C. Green J. B. Hauske S. J. Emberson J. R. (2023). Empagliflozin in patients with chronic kidney disease. N. Engl. J. Med. 388 (2), 117–127. 10.1056/NEJMoa2204233 36331190 PMC7614055

[B30] Udell J. A. Jones W. S. Petrie M. C. Harrington J. Anker S. D. Bhatt D. L. (2022). Sodium glucose cotransporter-2 inhibition for acute myocardial infarction: JACC Review topic of the week. J. Am. Coll. Cardiol. 79, 2058–2068. 10.1016/j.jacc.2022.03.353 35589167 PMC8972442

[B31] Wanner C. Inzucchi S. E. Lachin J. M. Fitchett D. von Eynatten M. Mattheus M. (2016). Empagliflozin and progression of kidney disease in type 2 diabetes. N. Engl. J. Med. 375 (4), 323–334. 10.1056/NEJMoa1515920 27299675

[B32] Webster A. C. Nagler E. V. Morton R. L. Masson P. (2017). Chronic kidney disease. Lancet 389 (10075), 1238–1252. 10.1016/S0140-6736(16)32064-5 27887750

[B33] Wiviott S. D. Raz I. Bonaca M. P. Mosenzon O. Kato E. T. Cahn A. (2019). Dapagliflozin and cardiovascular outcomes in type 2 diabetes. N. Engl. J. Med. 380 (4), 347–357. 10.1056/NEJMoa1812389 30415602

[B34] Zhou L. Cryan E. V. D’Andrea M. R. Belkowski S. Conway B. R. Demarest K. T. (2003). Human cardiomyocytes express high level of Na+/glucose cotransporter 1 (SGLT1). J. Cell. Biochem. 90, 339–346. 10.1002/jcb.10631 14505350

[B35] Zhuo M. Li J. Buckley L. F. Tummalapalli S. L. Mount D. B. Steele D. J. R. (2022). Prescribing patterns of sodium-glucose cotransporter-2 inhibitors in patients with CKD: A cross-sectional registry analysis. Kidney360 3 (3), 455–464. 10.34067/KID.0007862021 35582176 PMC9034822

[B36] Zinman B. Wanner C. Lachin J. M. Fitchett D. Bluhmki E. Hantel S. (2015). Empagliflozin, cardiovascular outcomes, and mortality in type 2 diabetes. N. Engl. J. Med. 373 (22), 2117–2128. 10.1056/NEJMoa1504720 26378978

